# An improved method for generating axenic entomopathogenic nematodes

**DOI:** 10.1186/s13104-015-1443-y

**Published:** 2015-09-19

**Authors:** Shruti Yadav, Upasana Shokal, Steven Forst, Ioannis Eleftherianos

**Affiliations:** Insect Infection and Immunity Lab, Department of Biological Sciences, Institute for Biomedical Sciences, The George Washington University, Washington DC, USA; Department of Biological Sciences, University of Wisconsin-Milwaukee, Milwaukee, WI USA

**Keywords:** *Drosophila*, *Steinernema*, *Xenorhabdus*, Entomopathogenic nematode, Parasitism, Infection, Immunity

## Abstract

**Background:**

*Steinernema carpocapsae* are parasitic nematodes that invade and kill insects. The nematodes are mutualistically associated with the bacteria *Xenorhabdus nematophila* and together form an excellent model to study pathogen infection processes and host anti-nematode/antibacterial immune responses. To determine the contribution of *S. carpocapsae* and their associated *X. nematophila* to the successful infection of insects as well as to investigate the interaction of each mutualistic partner with the insect immune system, it is important to develop and establish robust methods for generating nematodes devoid of their bacteria.

**Findings:**

To produce *S. carpocapsae* nematodes without their associated *X. nematophila* bacteria, we have modified a previous method, which involves the use of a *X. nematophilarpoS* mutant strain that fails to colonize the intestine of the worms. We confirmed the absence of bacteria in the nematodes using a molecular diagnostic and two rounds of an axenicity assay involving appropriate antibiotics and nematode surface sterilization. We used axenic and symbiotic *S. carpocapsae* to infect *Drosophila melanogaster* larvae and found that both types of nematodes were able to cause insect death at similar rates.

**Conclusion:**

Generation of entomopathogenic nematodes lacking their mutualistic bacteria provides an excellent tool to dissect the molecular and genetic basis of nematode parasitism and to identify the insect host immune factors that participate in the immune response against nematode infections.

## Findings

### Background

The entomopathogenic (or insect pathogenic) nematodes *Steinernema carpocapsae* form an obligate mutualistic association with the Gram-negative bacteria *Xenorhabdus nematophila* in the family Enterobacteriaceae [1]. The *S. carpocapsae*–*X. nematophila* nematode–bacteria complex has emerged as a biological control agent of diverse insect pest species [2, 3]. Nematodes of the infective juvenile (IJ) stage, which is the only stage that is able to survive outside of the host, enter insects through natural openings or by piercing the body wall [4, 5]. Once inside the insect body cavity, the IJ releases the bacteria into the hemolymph where they divide exponentially and produce a wide range of toxins and virulence factors that result in insect death [6]. The nematodes feed on the bacterial biomass, and insect tissues, and nematode reproduction continues over 2–3 generations until the nutrient status of the cadaver deteriorates whereupon progeny IJs colonized with *X. nematophila* disperse in search of new hosts. Transmission of mutualistic bacteria by IJ nematodes to the insect is essential for the nematodes to parasitize insects successfully and to reproduce [7, 8]. Instead the nematodes provide nutrients to their associated bacteria by permitting access to the insect host [9].

A major advantage of this mutualistic–pathogenic complex is that *S. carpocapsae* nematodes, like other entomopathogenic nematodes, are viable in the absence of their mutualistic *X. nematophila* bacteria (axenic nematodes) [10]. Consequently, each partner in the mutualistic relationship can be separated and studied in isolation or in combination enabling host immune responses to be studied against each partner separately, and against both partners together [11–14]. Therefore, this extremely efficient relationship is an excellent model for simultaneously investigating the molecular and functional basis of anti-nematode and anti-bacterial immune responses in the insect host, as well as for analyzing factors that promote nematode parasitism and bacterial pathogenicity [15, 16].

Here we describe a modification of a previous protocol for generating *S. carpocapsae* entomopathogenic nematodes without the presence of their *X. nematophila* bacteria [17]. A recent study has reported in vivo and in vitro laboratory procedures for maintaining entomopathogenic nematodes and a method that precludes the use of antibiotics for generating nematodes free of their mutualistic bacteria [18]. To generate *S. carpocapsae* axenic nematodes, we use *X. nematophila* mutant bacteria that support the growth of their nematode hosts but are not naturally acquired by the parasites [19]. This method can be readily used in combination with a wide range of molecular/genetic and physiological techniques to study nematode parasitism and humoral/cellular anti-nematode immune reactions in model insects as well as in insects of agricultural or medical importance.

## Methods

The mutant bacteria *X. nematophila* Δ*rpoS* [17] were used for generating *S. carpocapsae* axenic nematodes. For inoculation of liquid cultures, the bacteria were grown in 2 ml Luria–Bertani (LB) broth (BD Difco), overnight at 30 °C in a shaker-incubator at 220 rpm. Δ*rpoS* bacterial cultures were supplemented with 50 μg/ml ampicillin (Fisher Scientific) and 30 μg/ml kanamycin (Corning) because Δ*rpoS* mutants contain a kanamycin cassette and an ampicillin resistant plasmid [17]. An aliquot of 250 μl of the overnight culture was added to fresh 5 ml LB and the mix was incubated at 30 °C with shaking for 22–24 h.

For preparation of 20 oily agar plates, we mixed 300 ml of growth media containing 2.4 g of nutrient broth (BD Difco), 4.5 g of bacteriological agar (Amresco), 1.5 g of yeast extract (Amresco) and 267 ml of distilled water. The mix was autoclaved and the following components were then added to the media: 3 ml of 0.98 M MgCl_2_, 28.8 ml of 7.3 % sterile corn syrup and 1.2 ml of sterile corn oil. After autoclaving the solution, ampicillin and kanamycin were added to the media and the mix was stirred and then poured into one side of the bi-plates. The *X. nematophila* Δ*rpoS* bacterial culture (100 μl) was pipetted onto the oily agar media and spread evenly with a sterile spreader. The plates were incubated at 30 °C for 24 h.

For nematode surface sterilization, *S. carpocapsae* worms resuspended in 1 ml of sterile water were pipetted into a 1.5 ml Eppendorf tube and the solution was spun at 13,000 rpm for 10 s at room temperature to obtain a concentrated nematode pellet. The supernatant was discarded and 1 ml of freshly prepared 1 % bleach solution was added to the nematode pellet. The suspension was mixed well and the nematode pellet was washed in 1 ml of sterile distilled water to remove the bleach residue. The washing step was repeated five times. The nematode pellet was resuspended in appropriate volume of distilled water and the number of nematodes was counted using a stereoscope.

For nematode collection, 500–700 surface-sterilized symbiotic *S. carpocapsae* nematodes were transferred to the bacterial plates that were kept in a cabinet lined with moist paper towels at room temperature. After approximately 10 days, the plates were observed under a stereoscope to monitor the age and condition of the nematodes. When the IJ stage was reached in approximately 2–3 weeks, water traps were prepared and first round nematodes (Round 1) were collected in cell culture flasks [17]. To ensure that all *S. carpocapsae* nematodes were free of mutualistic *X. nematophila* bacteria, we used surface-sterilized Round 1 worms to repeat the same process, and second round nematodes (Round 2) were collected.

For testing the presence or absence of *X. nematophila* bacterial cells in *S. carpocapsae* nematodes, 1 ml of sterile water containing highly concentrated nematodes (approximately 50 worms/μl) was pipetted into a 1.5 ml Eppendorf tube. We included surface-sterilized and non surface-sterilized nematodes from Round 1 (Round 1: SS and Round 1) and Round 2 (Round 2: SS and Round 2) as well as symbiotic *S. carpocapsae* nematodes as controls. The solution was centrifuged at 13,000 rpm for 10 s at room temperature. The supernatant was discarded and the nematode pellet was homogenized using a small plastic pestle. The nematode homogenate was plated onto LB agar plates (one plate per treatment), which were incubated at 30 °C for 24 h. Growth of bacterial colonies on the plates indicated that *S. carpocapsae* nematodes contained their mutualistic *X. nematophila* bacteria (symbiotic nematodes) whereas lack of bacterial colonies on the plates indicated the absence of bacteria in the nematodes (axenic nematodes). The experiment was repeated at least five times.

For diagnosing the axenicity status of *S. carpocapsae* IJ nematodes, 100 μl pellets containing worms from Round 1 and Round 2 of the axenicity assay, and symbiotic nematodes (as positive control) were used. The nematodes were crushed using a pestle and DNA was extracted using the DNeasy Blood and Tissue Kit (Qiagen) by following the manufacturer’s instructions. *X. nematophilaXptA2* gene specific set of primers (Forward: GCCTGGAAAGAGTGGACGAA, Reverse: GTAAGACCAAGGGGCACTCC) were used for PCR amplification using the HotMasterMix (5 Prime) [20]. The cycling program was as follows: 95 °C for 2 min, 34 cycles of 95 °C for 30 s, annealing temperature of 61 °C for 1 min and 73 °C for 1 min followed by 72 °C for 10 min. The samples were viewed on a 1.5 % agarose gel to determine the presence or absence of *XptA2* bands.

For infection of *Drosophila melanogaster* larvae with *S. carpocapsae* IJ nematodes, 100 μl of 1.5 % agarose gel were added to the wells of a 96-well microtitre plate. The agarose was allowed to cool for 3 h prior to use. Third instar *D. melanogaster* larvae (Oregon strain) were transferred onto a Whatman filter paper using a fine soft bristle paintbrush and then washed by pipetting a small drop of sterile water to remove any food debris from their surface. Prior to infection, the symbiotic IJ nematodes were washed with sterile distilled water and the axenic nematodes were surface sterilized using bleach and then washed with distilled water, as mentioned above. The washed nematodes were then suspended in fresh sterile distilled water. A drop of 10 μl of water containing 100 symbiotic or axenic *S. carpocapsae* IJ nematodes and a single *D. melanogaster* larva were added to each well of the microtitre plate. Treatment with sterile distilled water (10 μl) served as control. Each row of the 96-well plate was covered with a strip of PCR clear film (Eppendorf) and holes were poked to allow air circulation. Thirty larvae were used per treatment and fresh batches of nematodes for each experiment. The results represent at least three independent experiments conducted on three different days. Values were expressed as means ± the standard deviation. Comparisons between survival curves was performed using a long-rank (Mantel–Cox) test in GraphPad Prism 5.0 software.

## Results

**Fig. 1:**
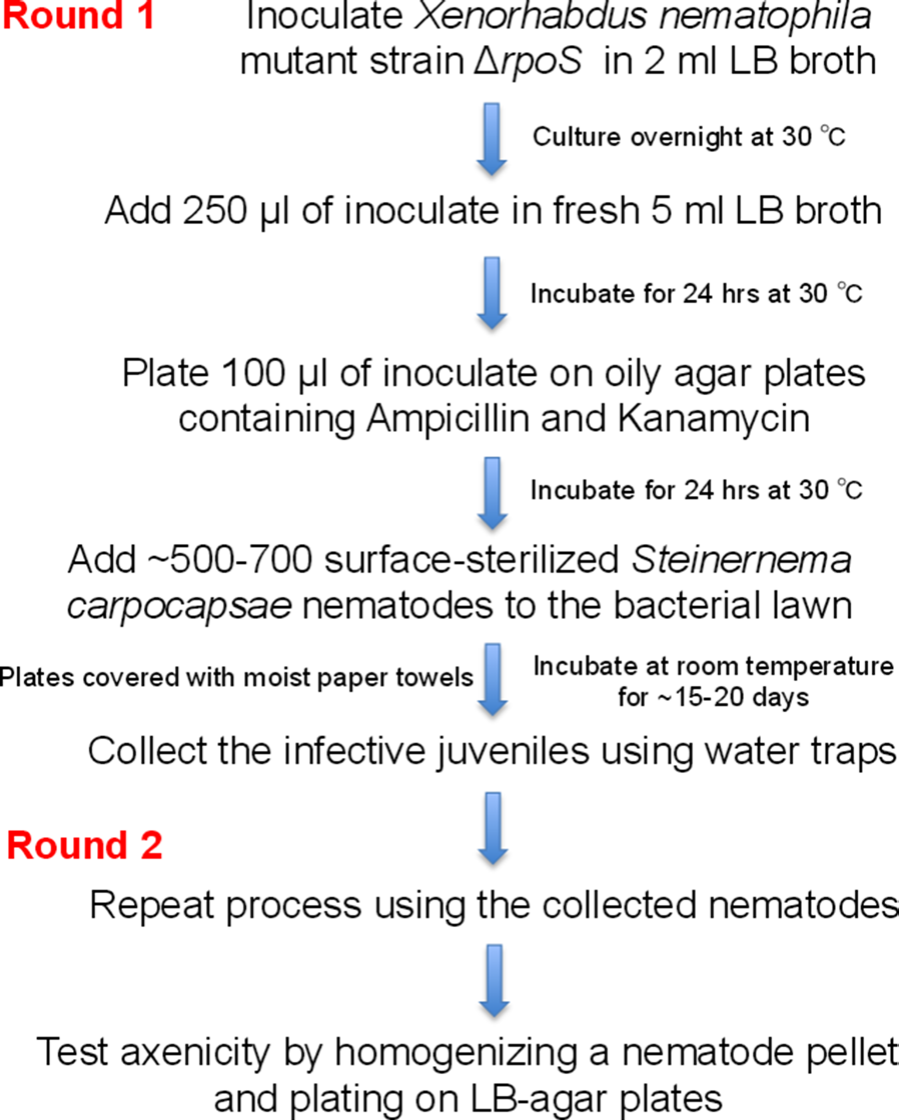
Flow diagram of the method for obtaining axenic nematodes. *Xenorhabdus nematophila* Δ*rpoS* mutant bacteria are grown overnight and then subcultured before plating on oily agar plates containing antibiotics. Surface-sterilized *Steinernema carpocapsae* nematodes are transferred to the plates covered by the mutant bacteria and after 3–4 weeks infective juvenile progeny are collected in water-traps. These steps consist the first round (Round 1) of the method. The entire procedure is repeated (Round 2) and the newly emerged nematodes are tested for the presence or absence of mutualistic *X. nematophila* bacteria

**Fig. 2:**
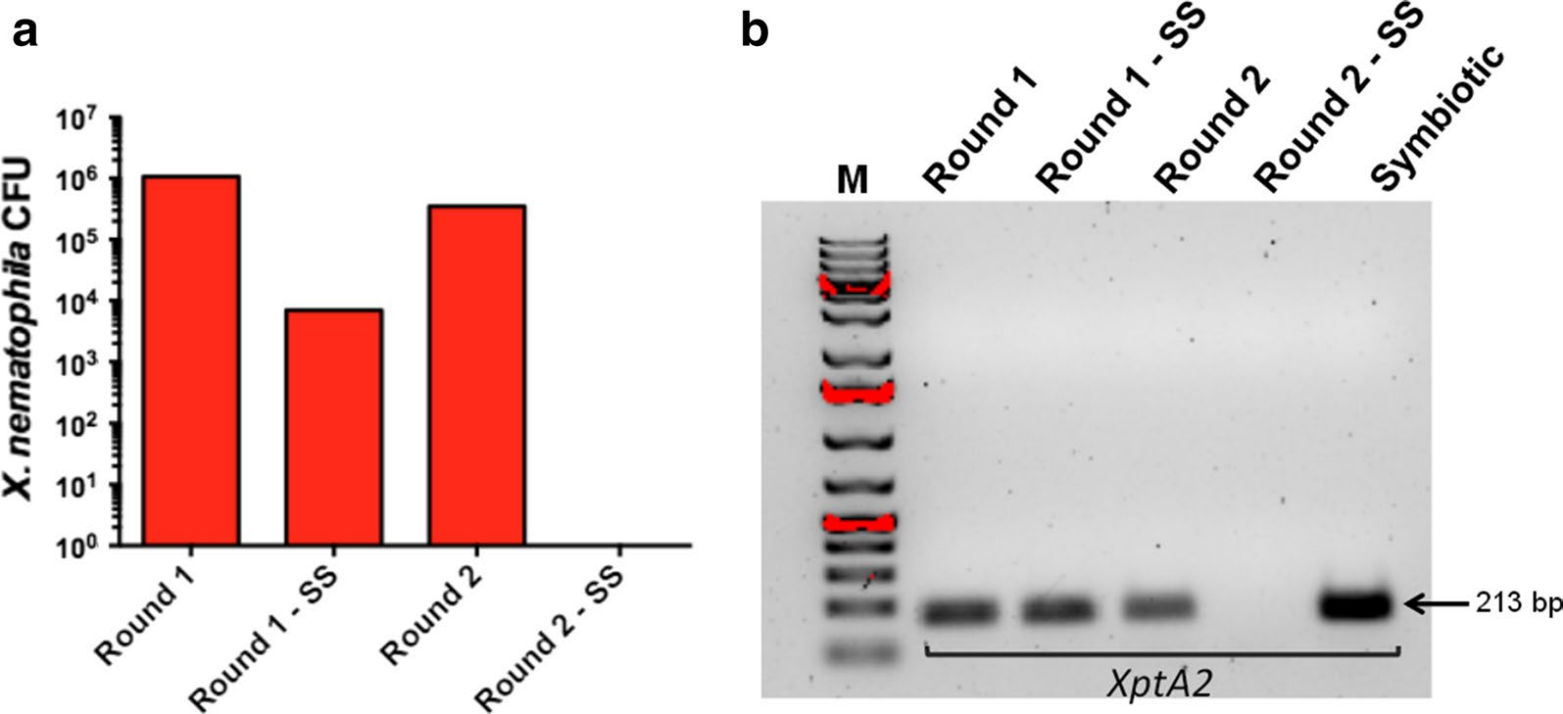
Validation of nematode axenicity status. **a** To estimate the presence of *Xenorhabdus nematophila* bacterial cells in *Steinernema* *carpocapsae* nematodes, a nematode pellet is homogenized and the homogenate is spread onto agar plates. The absence of *X. nematophila* colonies on the plates denotes that the nematodes are free of bacterial cells. Bacterial colony forming units (CFU, log scale) are shown in Round 1 and Round 2 of the axenicity assay. *SS* surface sterilized nematodes. **b** Diagnostic PCR for detecting the presence or absence of *X. nematophila* bacteria in surface-sterilized or non-surface-sterilized *S. carpocapsae* nematodes that were subjected to a single round of the axenicity assay (Round 1 and Round 1: SS) or two rounds of the procedure (Round 2 and Round 2: SS). Symbiotic nematodes served as control. The size of the PCR amplified *X. nematophila* *XptA2* gene is indicated

The protocol described here reports a modified method for generating *S. carpocapsae* entomopathogenic nematodes lacking their *X. nematophila* mutualistic bacteria (Fig. 1). Using a standard plating technique, we have found that completion of the first round of the process results in nematodes containing their *X. nematophila* bacteria (Round 1, Fig. 2a). We have also found that surface sterilized nematodes subjected to the first round of the axenicity assay still contained *X. nematophila* bacteria (Round 1: SS, Fig. 2a). To eliminate all *X. nematophila* cells from *S. carpocapsae* nematodes, we repeated the entire method using the nematodes that were generated from Round 1. Repeating the method cleared the nematodes from their associated bacteria, which was further confirmed by surface sterilization of the worms (Round 2: SS, Fig. 2a) leading to the generation of *S. carpocapsae* axenic nematodes. Importantly, we found that addition of the nematode sterilization step was crucial for removing the *X. nematophila* cells from the surface of the worms (Round 2, Fig. 2a).

Using a PCR diagnostic method, we amplified a 213 bp *X. nematophilaXptA2* gene sequence from DNA samples extracted from bacteria associated with *S. carpocapsae* nematodes, which had been generated through Round 1, Round 1: SS and Round 2 of the axenicity assay. However, there was no amplification of *XptA2* sequences from Round 2: SS samples (Fig. 2b). These results suggested that the axenicity assay was efficient in clearing *X. nematophila* bacteria from *S. carpocapsae* nematodes; therefore resulting in the generation of axenic worms.

**Fig. 3:**
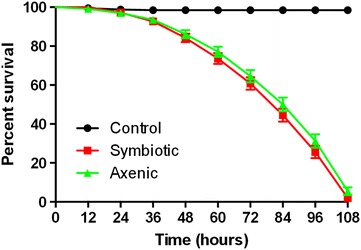
Survival results for *Drosophila* larvae infected by *Steinernema* nematodes. *Drosophila melanogaster* Oregon third instar larvae were infected by axenic (lacking *Xenorhabdus nematophila* bacteria) or symbiotic (containing *X. nematophila* bacteria) *Steinernema carpocapsae* infective juvenile nematodes. Treatment with sterile distilled water served as negative control. Survival was monitored every 12 h. Results showed that axenic and symbiotic nematodes were equally pathogenic to *D. melanogaster* larvae (*P* > 0.1, Log-Rank Test; GraphPad Prism 5)

We have used the symbiotic and axenic *S. carpocapsae* nematodes in infection assays to assess their potency against *D. melanogaster* larvae. We found that infection of *D.melanogaster* third instar larvae with the two types of nematodes resulted in insect death within 4.5 days post challenge with the parasites. Interestingly, we found no significant differences between the survival curves of fruit fly larvae following infection with axenic or symbiotic worms (Fig. 3; *P* > 0.1, Log-Rank Test).

## Discussion

Entomopathogenic nematodes are widely used in crop protection for the effective control of soil-borne insect pests, and they are excellent models for dissecting the molecular and genetic basis of parasitism and host anti-nematode immune function [2, 13]. Because the nematode–bacteria complex dissociates once inside the insect [21], it is possible that the host activates distinct immune responses against each mutualistic partner. There is also potential that the nematodes and their associated bacteria employ different strategies to evade or suppress the host immune system. To investigate these possibilities it is important to use robust methods for generating nematode parasites lacking their mutualistic bacteria (axenic nematodes).

Here we report a modified and improved method for the production and experimental use of *S. carpocapsae* nematodes without their mutualistic *X. nematophila* bacteria. The current method is based on a previously published procedure [17]. The relationship between *S. carpocapsae* and *X. nematophila* is highly specific and nematodes will only maintain mutualistic associations with their cognate bacteria [1]. Therefore, to produce *S. carpocapsae* nematodes without *X. nematophila* bacteria we used a *X. nematophila* strain containing a mutation in the *rpoS* gene that codes for the transcription factor *sigma*(*S*), which regulates survival of the bacteria, resistance to stress and interactions with their nematode host [19]. *X. nematophilarpoS* mutants have been shown previously to abolish the ability of the bacteria to colonize the intestine of *S. carpocapsae* IJ, which negates the mutualistic relationship between the two partners [19]. A recently described method involves the inoculation of agar plates with surface-sterilized eggs and does not require the addition of antibiotics [18]. The main differences between the current protocol and previous methods is the use of surface-sterilized IJ nematodes and the incorporation of antibiotics into the media to generate axenic worms. We consider the latter as an important step toward preventing the growth of other unwanted bacteria or fungal contamination in the nematode preparations.

We have used 1 % bleach solution for surface sterilization of the nematodes. This method eliminates all *X. nematophila* bacteria from the surface of the worms. The IJ stage is the developmentally arrested stage of most entomopathogenic nematodes and is analogous to the *Caenorhabditis elegans* dauer stage and the developmentally arrested infective third stage larva (L3) of many important parasitic nematodes [22]. During the IJ stage the nematode mouth is closed [23, 24], thus treatment with bleach eliminates only the bacterial cells that are present on the surface of the worms without affecting nematode infectivity.

We have found no differences in pathogenicity between axenic and symbiotic nematode infections of *D. melanogaster* larvae. Given that *X. nematophila* bacteria are potent pathogens of fruit flies and other insects [6, 25], we would have expected to find increased pathogenicity of symbiotic nematodes toward *D. melanogaster* larvae compared to infections with axenic worms. The reason for this unexpected result is currently unknown and requires further investigation. Previous studies involving *D. melanogaster* and *Manduca sexta* larvae have reported that *Heterorhabditis bacteriophora* nematodes without their mutualistic *Photorhabdus luminescens* bacteria are less pathogenic than symbiotic nematodes [11, 12]. However, another study has shown that *S. carpocapsae* IJ with or without their mutualistic *X. nematophila* bacteria are equally pathogenic to *Spodopteraexigua* larvae in laboratory and greenhouse experiments [17], and we have recently found that *H. bacteriophora* symbiotic nematodes are as pathogenic as axenic worms following infection of *D. melanogaster* adult flies [13]. It is worth noting that all infection experiments in the current study used *D. melanogaster* Oregon strain larvae whereas infection assays in previous investigations used Cinnabar brown strain larvae [11, 14]. We have recently found that different *D. melanogaster* wild-type strains can exhibit strong variation in their immune response against microbial infections [26].

Our current results suggest that the presence of *X. nematophila* mutualistic bacteria in *S. carpocapsae* nematodes is probably not imperative for the ability of the worms to efficiently infect and kill *D. melanogaster* wild-type larvae. Therefore it is possible that *X. nematophila* contribute to the reproductive fitness of *S. carpocapsae* nematodes without providing an additional advantage to the pathogenicity of the worms [19]. Alternatively, the nematodes may produce certain molecules that could enhance pathogenicity or molecules that could potentially mask the activity of *X. nematophila* virulence factors that are secreted during infection of insects [27, 28]. It is also possible that migration and constant movement of *S. carpocapsae* nematodes, even in the absence of their mutualistic bacteria, within *D. melanogaster* larvae could result in severe physical damage of vital insect tissues and organs, which could ultimately lead to insect death.

## Conclusion

Here we describe a modified method for the generation of parasitic nematodes without their mutualistic bacteria. This method involves the completion of two rounds of an axenicity protocol, the use of appropriate antibiotics and nematode surface sterilization treatment to eliminate the presence of bacterial cells on the surface of the worms. This method will promote studies on the molecular basis of nematode parasitism, host anti-nematode immunity and host-microbial mutualism, and it will assist in the identification of nematode genes that participate in these important biological processes.

## Abbreviations

IJ: infective juvenile
LB: Luria–Bertani
PCR: polymerase chain reaction
PBS: phosphate buffered saline
